# Sizes of actin networks sharing a common environment are determined by the relative rates of assembly

**DOI:** 10.1371/journal.pbio.3000317

**Published:** 2019-06-10

**Authors:** Adrien Antkowiak, Audrey Guillotin, Micaela Boiero Sanders, Jessica Colombo, Renaud Vincentelli, Alphée Michelot

**Affiliations:** 1 Aix Marseille Univ, CNRS, IBDM, Turing Centre for Living Systems, Marseille, France; 2 Unité Mixte de Recherche (UMR) 7257, Centre National de la Recherche Scientifique (CNRS) Aix-Marseille Université, Architecture et Fonction des Macromolécules Biologiques (AFMB), Marseille, France; Utrecht University, NETHERLANDS

## Abstract

Within the cytoplasm of a single cell, several actin networks can coexist with distinct sizes, geometries, and protein compositions. These actin networks assemble in competition for a limited pool of proteins present in a common cellular environment. To predict how two distinct networks of actin filaments control this balance, the simultaneous assembly of actin-related protein 2/3 (Arp2/3)-branched networks and formin-linear networks of actin filaments around polystyrene microbeads was investigated with a range of actin accessory proteins (profilin, capping protein, actin-depolymerizing factor [ADF]/cofilin, and tropomyosin). Accessory proteins generally affected actin assembly rates for the distinct networks differently. These effects at the scale of individual actin networks were surprisingly not always correlated with corresponding loss-of-function phenotypes in cells. However, our observations agreed with a global interpretation, which compared relative actin assembly rates of individual actin networks. This work supports a general model in which the size of distinct actin networks is determined by their relative capacity to assemble in a common and competing environment.

## Abstract 2

A biomimetic assay using polystyrene beads compares the rates of actin assembly on linear and branched networks, revealing how the size of rival actin networks in cells is regulated by their relative capacity to assemble in a common environment.

## Introduction

Eukaryotic cells assemble a range of filamentous actin (F-actin) structures from actin monomers (globular actin [G-actin]) to accomplish diverse processes. Actin assemblies may be formed generally through two different mechanisms: (1) branched networks of actin filaments, for which actin filaments are nucleated and elongate from the side of pre-existing ones by a 7-subunit complex called the actin-related protein 2/3 (Arp2/3) complex and (2) linear networks of actin filaments, for which actin filaments are capped at their dynamic barbed ends by a homo-dimer of formin and elongate processively by the insertion of actin monomers [1]. In addition, different actin networks have distinct geometrical organizations, sizes, dynamics, and mechanical properties, which are adapted to their precise function. These parameters are tightly controlled by specific accessory proteins. The accessory proteins may have one or multiple effects on actin filaments, which may include nucleating new filaments from free actin monomers, cross-linking filaments into specific geometries, controlling elongation rates, and severing and disassembling actin filaments [2]. The presence of all of these variables within a dynamic cell raises a number of challenges to our understanding of actin biology.

The size of actin networks is controlled by the balance between their specific rates of actin filament nucleation, rates of assembly at actin filament barbed ends, and rates of network disassembly. Originally, it was thought that each cellular actin network was assembling independently of the others due to the presence of a large and unlimited pool of actin monomers. As a consequence, this original model proposed that rates of actin polymerization and disassembly were constant and that the size of each actin network was mainly determined by the rate of actin nucleation. However, recent studies challenged this idea and demonstrated that different actin networks in cells were in tight competition for a limited pool of actin monomers [3–5]. This discovery of homeostatic actin networks suggests that beyond the regulation of actin nucleation, the growth rate and size of actin networks was also controlled by the concentration of polymerizable actin left in a competitive environment. For instance, cells in which the Arp2/3 complex activity is diminished, the disappearance of branched networks is tightly correlated with the assembly of an unusually high number of formin-dependent actin cables [3,6]. Conversely, formin inhibition does not change the level of F-actin but promotes the assembly of Arp2/3-branched networks in cells. With this new model, any growth of an actin network beyond its usual extent partially depletes the amount of G-actin that is available in the cytoplasm and therefore reduces the size of other actin networks. This concept was recently formalized theoretically under the name of global treadmilling [7].

The actin accessory protein profilin, which tightly binds to G-actin, was demonstrated to regulate F-actin network homeostasis by favoring the formin assembly pathway over the Arp2/3 complex assembly pathway [6,8]. Control of profilin expression is a powerful mechanism to regulate the formation of dynamic actin-rich protrusions and collective cell migration [9]. Such an observation suggests that fine-tuning the activity or expression of actin accessory proteins is an efficient and physiological mechanism for cells needing to reorganize rapidly their actin cytoskeleton. Besides profilin, most other actin accessory proteins have been shown to impact globally the organization of the actin cytoskeleton. This is the case for cultured cells [10,11] as well as in more complex processes such as epithelial-to-mesenchymal transitions [12]. However, how specific factors modulate globally the organization of the actin cytoskeleton in a homeostatic environment is not yet clear.

Understanding the global impact of an accessory protein is a challenge, as most proteins have multiple effects on actin assembly. For example, actin-depolymerizing factor (ADF)/cofilin binds to G-actin as well as to F-actin, with multiple effects on actin assembly, disassembly, and monomer recycling [2,13,14]. Furthermore, understanding how the activity of these accessory proteins modulates branched versus linear network assembly is confounded by the fact that they have different consequences on the formin and Arp2/3 assembly pathways. To circumvent this problem, the assembly of both actin assembly pathways were reconstituted in this study from a minimal number of essential components in a shared experimental environment in which the steady state is rapidly reached. Although both types of actin networks assembled independently, this system enabled us to test and compare side-by-side the effect of various families of proteins on both actin assembly pathways. Our results indicate that all actin accessory proteins potentially impact actin assembly pathways differently. This work demonstrates that the interpretation of these differences in the context of a competitive environment provides a general explanation of how the size of linear and branched networks is balanced in cells.

## Results

### Reconstitution of actin-based motility from WASp and formin-coated beads in a common environment

Previous studies reconstituted separately Arp2/3-based and formin-based actin motilities in vitro [15–17]. Typical assays use polystyrene microbeads coated by a nucleation-promoting factor of the Arp2/3 complex or coated by formins in an environment in which actin filament elongation is funneled at the bead surface to generate propulsive forces. Arp2/3-based actin motility is characterized by the assembly of dense tails of branched actin networks, in which beads are pushed by the continuous elongation of uncapped actin filament barbed ends at their surface [15]. Formin-based actin motility is characterized by the assembly of dense cables of linear actin filaments, in which beads are pushed by the processive elongation of actin filaments capped at their barbed ends by the formins [16,17].

**Fig 1:**
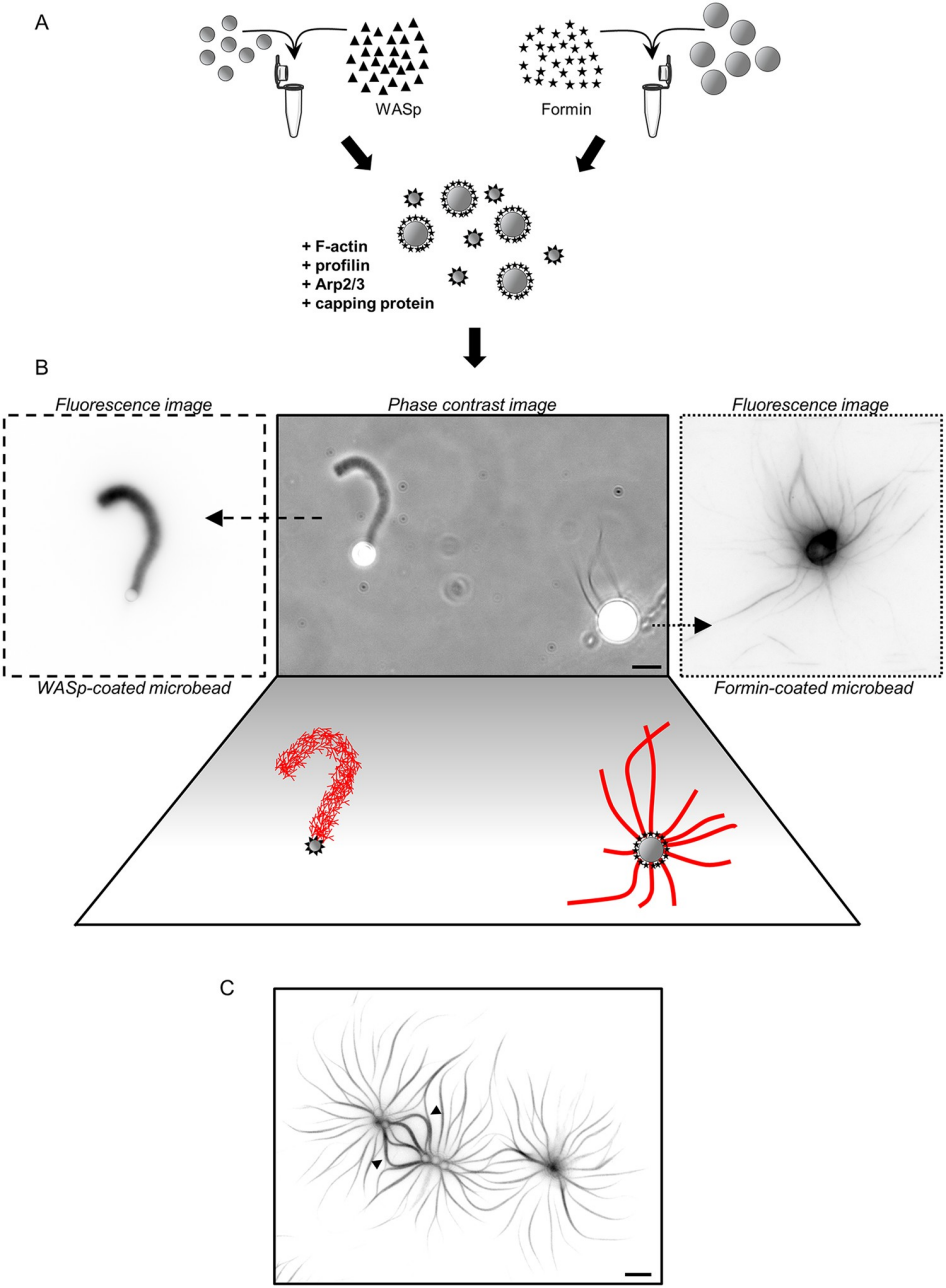
Simultaneous assembly of branched and linear networks of actin filaments in vitro. A. Schematic of experimental bead assay setup. B. Phase contrast and fluorescence snapshots of branched actin networks assembled around 2-μm diameter WASp-coated microbeads and linear actin networks assembled around 4-μm diameter formin-coated microbeads in the presence of fluorescent actin, Arp2/3 complex, profilin, and capping protein. Images were taken 30 min after the initiation of the experiment. Scale bar: 5 μm. C. Fluorescence snapshot of actin networks assembled around multiple formin-coated microbeads 45 min after the initiation of the experiment. Buckling events indicated by the black arrowheads. Scale bar: 10 μm. Arp2/3, actin-related protein 2/3; WASp, Wiskott–Aldrich syndrome protein.

Simultaneous Arp2/3-based and formin-based actin nucleation was reconstituted in vitro [8,18], but the set of essential accessory proteins that is required to obtain a simultaneous steady-state actin network assembly from Wiskott–Aldrich syndrome protein (WASp) and formin-coated beads has not yet been determined. Polystyrene microbeads of 2-μm diameter were coated with the budding yeast WASp ortholog (Las17p), and 4-μm diameter microbeads with the formin homology domains 1 and 2 (FH1-FH2) of a budding yeast formin, bud neck involved protein 1 (Bni1p) (Fig 1A). A sustained and organized actin assembly occurred immediately at the surface of the beads when they were mixed in a buffer solution containing solely 8 μM prepolymerized F-actin, 250 nM Arp2/3 complex, 1 μM capping protein, and 15 μM profilin (Fig 1B). Such experimental conditions were sufficient to generate actin networks of visibly distinct architectures. WASp-coated beads were propelled by assembling actin networks at rates that are comparable with other studies (typically up to 2 μm/min) [15,19], showing that the system is competent for actin-based motility and, more generally, for force generation. In the case of formin-coated beads, the beads often stalled rapidly, but force generation of actin networks is inferred from the buckling of some actin cables polymerizing at their surface [17] (Fig 1C). The use of a fluorescent actin reporter shows that the amount of polymer around both types of beads increases linearly from the beginning of the experiment and for several tens of minutes, suggesting a constant and measurable rate of actin assembly for all conditions tested (S1A Fig). Actin assembly rates on WASp-coated beads were systematically measured on comet tails after symmetry breaking, and experiments performed with submicron size beads, which undergo symmetry breaking more rapidly, gave similar results (S1B Fig). The design of this biomimetic system presents the advantage to compare the efficiency of actin assembly for two distinct assembly pathways in equivalent conditions.

### WASp/formin biomimetic assay recapitulates the effect of an inhibition of the Arp2/3 complex

**Fig 2:**
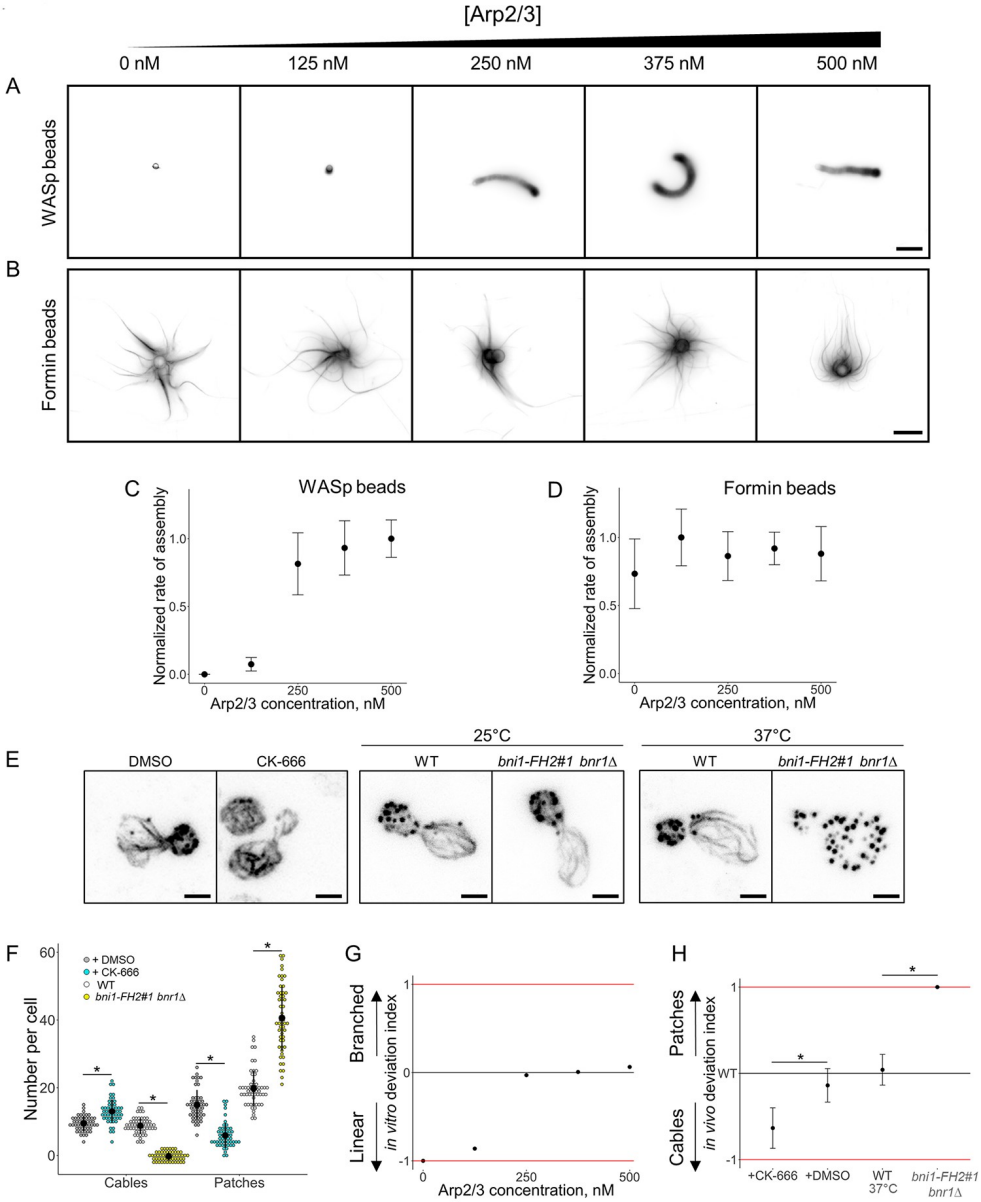
Modulation of the branched-to-linear actin network balance by the Arp2/3 complex. The underlying data can be found within S1 Data. A. Fluorescence snapshots of actin networks assembled around WASp-coated microbeads in the presence of fluorescent actin, profilin, capping protein, and variable concentrations of Arp2/3 complex. Images were taken 30 min after the initiation of the experiment. Scale bar: 5 μm. B. Fluorescence snapshots of actin networks assembled around formin-coated microbeads in the presence of fluorescent actin, profilin, capping protein, and variable concentrations of Arp2/3 complex. Images were taken 30 min after the initiation of the experiment. Scale bar: 5 μm. C. Quantification of (A). Rate of actin assembly around WASp-coated microbeads as a function of the Arp2/3 complex concentration, normalized to the maximum value. D. Quantification of (B). Rate of actin assembly around formin-coated microbeads as a function of the Arp2/3 complex concentration, normalized to the maximum value. E. Snapshots of the actin cytoskeleton organization in budding yeast cells fixed and labeled with fluorescent phalloidin, in the presence or in the absence of 200 μM CK-666 (left images), and for the formin defective mutant *bni1-FH2#1 bnr1*Δ at nonrestrictive temperature (25 °C; center images) or restrictive temperature (37 °C; right images). Scale bars: 2 μm. F. Quantification of (E). Average number of actin patches and cables per cell. G. In vitro deviation index, calculated as a function of the Arp2/3 complex concentration. This index compares how actin assembly rates around WASp and formin-coated beads deviate from a balanced situation in which both types of networks assemble optimally. H. In vivo deviation index, based on structures number, calculated in the presence of DMSO, 200 μM CK-666, at 37 °C for wild-type cells or at 37 °C for *bni1-FH2#1 bnr1*Δ cells. This index compares how the number of actin patches and cables deviate from the wild-type condition. Arp2/3, actin-related protein 2/3; CK-666, Arp2/3 complex inhibitor I; WASp, Wiskott–Aldrich syndrome protein.

We wanted to confirm that beyond visibly distinct actin architectures, both WASp and formin-coated beads were mimicking appropriately the assembly of branched and linear actin networks in vivo. We investigated first the sensitivity of actin network assembly in the biomimetic assay to variable concentrations of Arp2/3 complex. The concentrations of profilin and capping protein were fixed at values that are optimal for both branched and linear network assembly, and the concentration of Arp2/3 complex was varied between 0 and 500 nM (Fig 2A and 2B). As expected, increasing concentrations of Arp2/3 complex enhanced actin nucleation on WASp-coated beads and increased the rate of actin assembly on these beads (Fig 2C). However, the Arp2/3 complex is not expected to affect actin nucleation on formin-coated beads, and actin assembly on formin-coated beads was constant over a large range of Arp2/3 concentration (Fig 2D).

We next determined if the reconstitution mimics the effect of an Arp2/3 inhibition in vivo. Budding yeast is a convenient system for such comparison as only two spatially distinct actin networks are present during most of its cell cycle. The Arp2/3 assembly pathway is represented by the presence of endocytic actin patches, while the formin assembly pathway is represented mainly by the presence of spatially distinct actin cables and in a lesser proportion by the presence of a cytokinetic actin ring [10,20]. Addition of the Arp2/3 complex inhibitor I (CK-666) in fission yeast reduces dramatically the number of patches, while the number and size of cables increases [3]. We stabilized, labeled with fluorescent phalloidin, and imaged the actin cytoskeleton of budding yeast cells in the presence or absence of CK-666 (Fig 2E and S2A Fig). On average, 15 actin patches per cell were visible in control conditions, but their number decreased to 6 actin patches on average in the presence of CK-666 (Fig 2F). The quantification of the total patch intensity per cell indicates a similar tendency, with an actin patch intensity per cell reduced on average by 75% in the presence of CK-666 (S2B Fig). The situation is inversed for actin cables. The number of visible cables per cell increases from 9 to 13 in the presence of CK-666, and cables assembled per cell are on average 240% brighter with CK-666 than in control conditions (Fig 2F and S2B Fig). These results demonstrate a similar effect of CK-666 in budding yeast as in fission yeast. Conversely, inhibition of formins in the temperature sensitive yeast mutant *bni1-FH2#1 bnr1*Δ [21] increases the number of actin patches to 41, while no actin cables are visible anymore (Fig 2E and 2F).

A close comparison of the results from the biomimetic assay and in cells shows a correlated increase in branched network assembly. However, the Arp2/3-independant linear actin network assembly in the biomimetic assay does not match with the variation in cable assembly observed in cells. The reason is that if the biomimetic assay allows an understanding of how two different networks of actin filaments assemble when encountering similar conditions, it does not take into account how they may compete for a common pool of proteins as they do in cells [3,5]. This discrepancy imposed a second type of analysis of our results in order to evaluate the capacity of each actin network to assemble relatively to the other actin network. This comparison was made by defining a deviation index, which compares the efficiency of actin assembly for both branched and linear networks of actin filaments for a given biochemical condition in vitro. This index measures how actin assembly between branched and linear networks deviates from an equilibrium situation in which actin assembles equally well for both networks and is defined as follows:

$$In\;vitro\;deviation\;index\;(\left[ABP\right])=\frac{r_{branched(\left[ABP\right])}-\;r_{linear(\left[ABP\right])}}{r_{branched(\left[ABP\right])}+\;r_{linear(\left[ABP\right])}},$$

in which *r* is the rate of actin assembly for branched or linear networks for a given concentration of a given accessory protein (actin-binding protein [ABP]).

This index varies between the values −1 and +1, in which −1 represents an extreme case in which only formin-coated beads assemble actin networks in vitro; 0 represents a balanced situation in which formin-coated beads assemble as much actin as WASp-coated beads; and +1 represents the other extreme case in which only WASp-coated beads assemble actin networks in vitro (Fig 2G).

Similarly, an in vivo index comparing the deviation in the number of actin patches and cables in yeast cells is defined as follows:

$$In\;vivo\;deviation\;index(\left[CK666\right])=\frac{\frac{N_{patches,CK666}}{N_{patches,DMSO}}-\;\frac{N_{cables,CK666}}{N_{cables,DMSO}}}{\frac{N_{patches,CK666}}{N_{patches,DMSO}}+\;\frac{N_{cables,CK666}}{N_{cables,DMSO}}},$$

in which *N* is the number of actin patches and cables in the presence or in the absence of CK-666 (Fig 2H). Defining an in vivo deviation index based on the intensity actin patches and cables gave us similar results throughout this study (S2C Fig). The in vivo deviation index also varies between the values −1 and +1, in which −1 represents an extreme case in which only actin cables are visible in cells; 0 represents the wild-type equilibrium; and +1 represents the other extreme case in which only actin patches are detected.

A comparison of the in vitro and in vivo indexes (Fig 2G and 2H) indicates that an appropriate interpretation of the biomimetic assay is able to predict how the balance between Arp2/3 and formin networks is modified by a change of the Arp2/3 complex activity. Both indexes indicate trends, thus should not be compared quantitatively, as a value 0 for the in vivo index is equivalent to the wild-type condition, while a value 0 for the in vitro index is equivalent to a condition in which both linear and branched network assemble equally well relative to their respective optimal condition of assembly. A more thorough analysis would require the precise knowledge of the cytoplasmic concentrations of the proteins used in these assays.

### WASp/formin biomimetic assay recapitulates the biochemical effect of the actin assembly regulator profilin

Profilin is an established regulator for the homeostasis of actin networks [6,8,22]. Profilin binds tightly to ATP-G-actin (K_d_ ≈ 100 nM) and prevents the spontaneous nucleation and the elongation of actin filaments at their pointed ends [1]. Profilin also binds directly to the poly-L-proline domain of formin and enhances processive barbed end elongation rates. In contrast, profilin reduces nucleation and branching by the Arp2/3 complex [8]. Profilin also binds to actin filament barbed ends, competing with other barbed end interactors, therefore triggering a multitude of effects on actin filament barbed-end dynamics [22]. Profilin also contributes to actin recycling by promoting the exchange of adenosine diphosphate (ADP) to adenosine triphosphate (ATP) on actin monomers.

**Fig 3:**
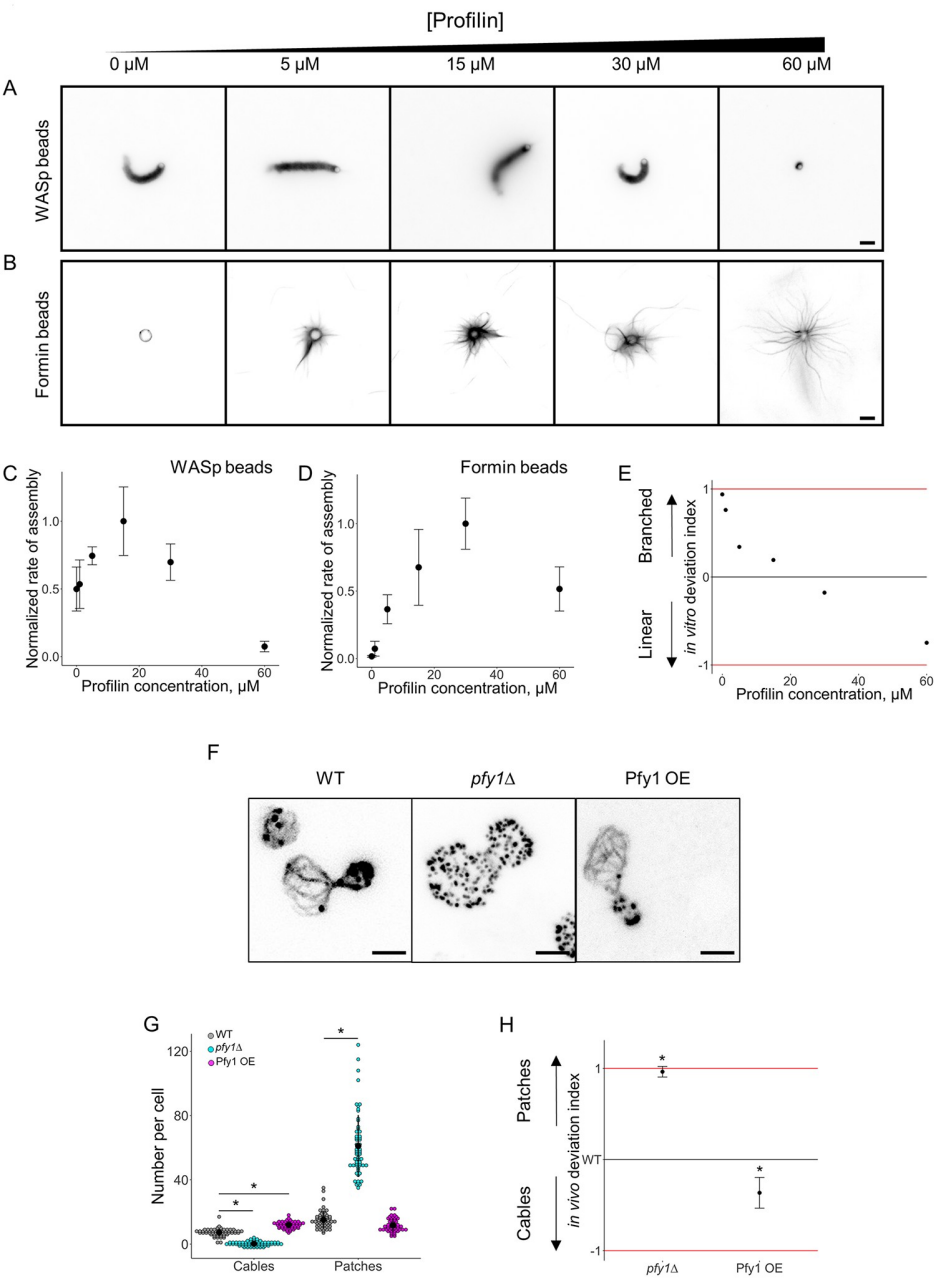
Modulation of the branched-to-linear actin network balance by profilin. The underlying data can be found within S1 Data. A. Fluorescence snapshots of actin networks assembled around WASp-coated microbeads in the presence of fluorescent actin, Arp2/3 complex, capping protein, and variable concentrations of profilin. Images were taken 30 min after the initiation of the experiment. Scale bar: 5 μm. B. Fluorescence snapshots of actin networks assembled around formin-coated microbeads in the presence of fluorescent actin, Arp2/3 complex, capping protein, and variable concentrations of profilin. Images were taken 30 min after the initiation of the experiment. Scale bar: 5 μm. C. Quantification of (A). Rate of actin assembly around WASp-coated microbeads as a function of the profilin concentration, normalized to the maximum value. D. Quantification of (B). Rate of actin assembly around formin-coated microbeads as a function of the profilin concentration, normalized to the maximum value. E. In vitro deviation index, calculated as a function of the profilin concentration. F. Snapshots of the actin cytoskeleton organization in wild-type, *pfy1*Δ, and Pfy1 overexpressing budding yeast cells fixed and labeled with fluorescent phalloidin. Scale bars: 2 μm. G. Quantification of (F). Average number of actin patches and cables per cell. H. In vivo deviation index for *pfy1*Δ and Pfy1 overexpressing cells. Arp2/3, actin-related protein 2/3; Pfy1, profilin; WASp, Wiskott–Aldrich syndrome protein.

Profilin was added in the biomimetic assay at various concentrations ranging from 0 to 60 μM (Fig 3A and 3B). Actin assembly curves for both types of networks displayed bell-shaped curves but with different characteristics (Fig 3C and 3D). Actin assembly and bead motility were modestly efficient for WASp-coated beads in the absence of profilin but were inhibited by the presence of large amounts of profilin (Fig 3C). On the contrary, actin assembly into cables was inefficient on formin-coated beads in the absence of profilin but efficient in the presence of up to 60 μM of profilin (Fig 3D). Another difference between these curves is that optimal conditions for actin assembly were reached at different concentrations of profilin, i.e., about 15 μM for WASp-coated beads and 30 μM for formin-coated beads (Fig 3C and 3D). These assays are performed at low bead densities, and we verified that the assembly of one type of actin network does not influence the assembly of the other (S3 Fig). Therefore, as for the Arp2/3 complex, the side-by-side comparison of these curves does not show intuitively whether branched network or linear network assembly is favored for a given concentration of profilin. Consequently, we plotted for each concentration of profilin the in vitro deviation index (Fig 3E). This deviation index varies between +0.94 in the absence of profilin to −0.75 for a high concentration of profilin, indicating nonambiguously the ability of this protein to switch actin assembly from branched networks on WASp-coated beads to cable assembly on formin-coated beads.

We compared our results with the effect of profilin in budding yeast cells [23]. Labeling of actin structures with fluorescent phalloidin indicated that profilin null (*pfy1*Δ) cells do not assemble any visible actin cables but assemble >5 times more actin patches than wild-type cells (Fig 3F and 3G and S2A Fig). On the contrary, overexpression of profilin reduced the number of actin patches from 15 in wild-type cells to 12 on average, while the number of actin cables increased from 7 to 12. As above, an in vivo deviation index is defined as follows:

$$In\;vivo\;deviation\;index=\frac{\frac{N_{patches,mutant}}{N_{patches,wild\;type}}-\;\frac{N_{cables,\;mutant}}{N_{cables,wild\;type}}}{\frac{N_{patches,mutant}}{N_{patches,wild\;type}}+\;\frac{N_{cables,mutant}}{N_{cables,\;wild\;type}}},$$

in which *N* is the number of actin patches and cables for wild-type or mutant cells. Calculation of the in vivo deviation index for wild-type, *pfy1*Δ, and profilin overexpressing cells shows a similar trend than the in vitro deviation index (Fig 3E and 3H), demonstrating again that the biomimetic assay and its analysis recapitulate the physiological effect of profilin in vitro.

### Effect of capping protein on Arp2/3-based and formin-based actin assembly pathways

Our previous results demonstrated that proteins such as Arp2/3 and profilin modulate branched and linear actin network assembly differently and, therefore, impact the balance between branched and linear networks of actin filaments in vivo. We followed for the rest of this study the hypothesis that any other protein implicated in actin assembly was also likely to affect actin assembly pathways differently and, therefore, impact the branched/linear actin network balance [7].

We first tested this hypothesis by focusing our attention on capping protein. Capping protein, which is a heterodimer of Cap1 and Cap2, binds tightly to the barbed end of actin filaments and inhibits their elongation [1]. On branched networks of actin filaments, capping protein limits the size of individual actin filaments, contributes to the densification of actin networks [24], and promotes filament nucleation by the Arp2/3 complex [25]. On linear networks of actin filaments, capping protein is able to bind simultaneously with formin to actin filament barbed ends [26,27]. However, this mutual binding is weak and enables rapid displacement of one by the other. As a consequence, capping protein competes with formin to bind actin filament barbed ends [28,29]. Capping protein also increases the steady-state concentration of monomeric actin in these assays, which can influence both pathways [30,31].

**Fig 4:**
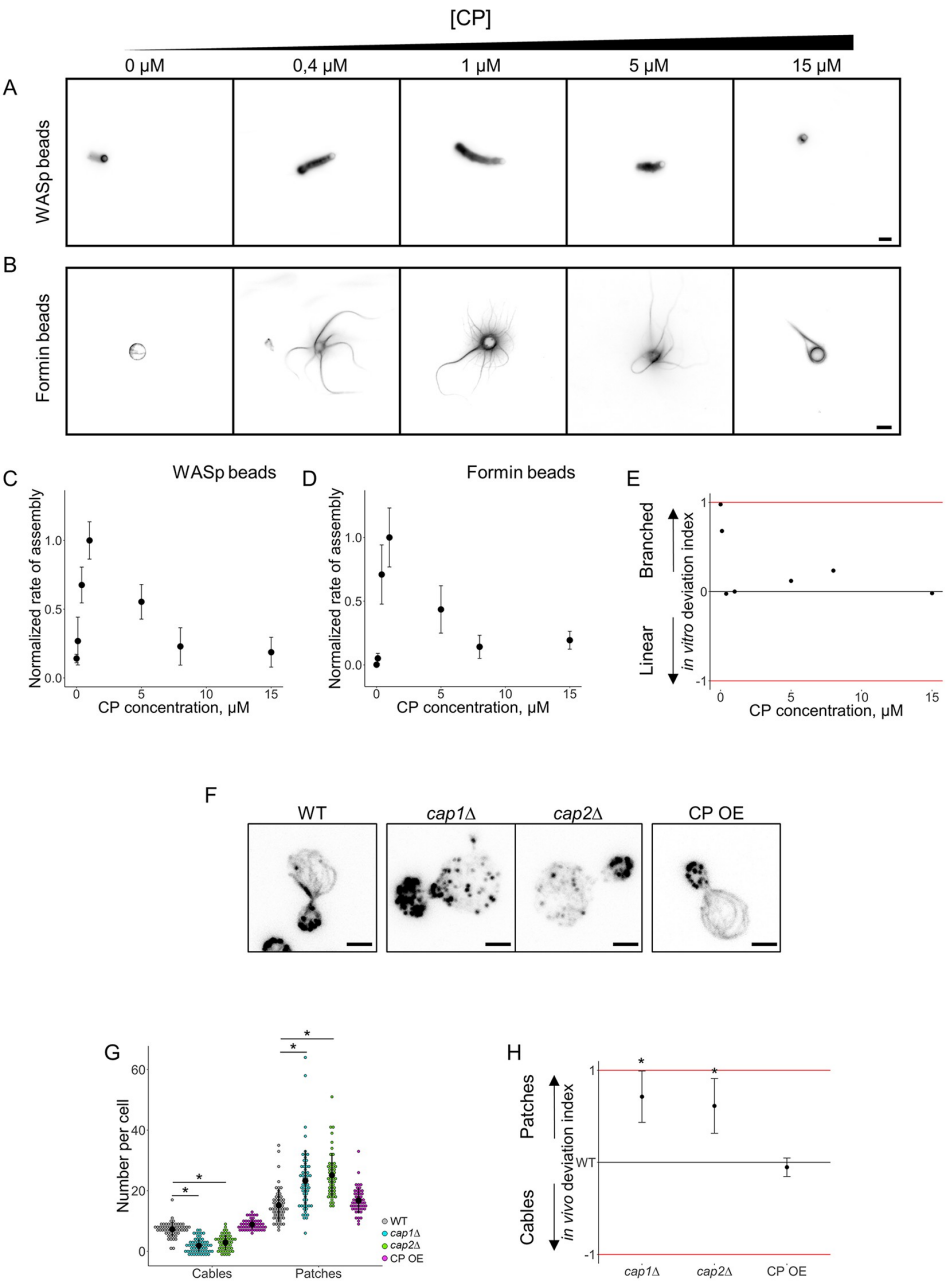
Modulation of the branched-to-linear actin network balance by capping protein. The underlying data can be found within S1 Data. A. Fluorescence snapshots of actin networks assembled around WASp-coated microbeads in the presence of fluorescent actin, Arp2/3 complex, profilin, and variable concentrations of capping protein. Images were taken 30 min after the initiation of the experiment. Scale bar: 5 μm. B. Fluorescence snapshots of actin networks assembled around formin-coated microbeads in the presence of fluorescent actin, Arp2/3 complex, profilin, and variable concentrations of capping protein. Images were taken 30 min after the initiation of the experiment. Scale bar: 5 μm. C. Quantification of (A). Rate of actin assembly around WASp-coated microbeads as a function of the capping protein concentration, normalized to the maximum value. D. Quantification of (B). Rate of actin assembly around formin-coated microbeads as a function of the capping protein concentration, normalized to the maximum value. E. In vitro deviation index, calculated as a function of the capping protein concentration. F. Snapshots of the actin cytoskeleton organization in wild-type, *cap1*Δ, *cap2*Δ, and capping protein overexpressing budding yeast cells fixed and labeled with fluorescent phalloidin. Scale bars: 2 μm. G. Quantification of (F). Average number of actin patches and cables per cell. H. In vivo deviation index for *cap1*Δ, *cap2*Δ, and capping protein overexpressing cells. Arp2/3, actin-related protein 2/3; WASp, Wiskott–Aldrich syndrome protein.

The concentration of capping protein was varied in the biomimetic assay between 0 and 15 μM (Fig 4A and 4B). As for profilin, both actin assembly pathways display bell-shaped curves with optimal concentrations of capping protein around 1 μM for both types of actin networks (Fig 4C and 4D). Actin network assembly occurred noticeably faster on WASp-coated beads than on formin-coated beads. At high concentration of capping protein, both networks were found to assemble equally well. Preincubation of proteins for 2 h at room temperature before introduction of the beads did not change our observations, indicating that steady-state actin assembly in these assays is reached rapidly from the disassembly of the pool of filamentous actin (S4A and S4B Fig). Overall, the determination of the in vitro deviation index for capping protein predicts that the assembly of branched networks is favored over linear networks for low concentrations of capping protein (<1 μM) and reaches a stable equilibrium between both structures for concentrations of capping protein above 1 μM (Fig 4E, S4A and S4B Fig). We compared these results with the effect of an absence of capping protein in yeast. As described previously, *cap1*Δ and *cap2*Δ cells have a similar phenotype because capping protein requires the expression of both subunits for its function [32]. Absence of capping protein in yeast was also correlated with higher amounts of actin polymer in cells, suggesting that capping protein is also important in cells to increase the concentration of polymerizable actin [33]. In both *cap1*Δ and *cap2*Δ cells, an elevated number of patches and very few actin cables were detectable (Fig 4F and 4G and S2A Fig). Remaining actin cables may be present due to the presence of other inhibitors of actin filament barbed-end elongation in yeast [34,35]. As a consequence, the in vivo deviation index was higher for both strains, as predicted by the biomimetic assay (Fig 4E and 4H). We also investigated the effect of an overexpression of capping protein in yeast cells. Overexpression was induced from a multicopy plasmid derived from the plasmid used for the endogenous expression and purification of a functional capping protein [36] (S5 Fig). Surprisingly, despite the strong overexpression of capping protein in these cells, their actin cytoskeleton appears to be normal (Fig 4F and 4G).

As low rates of actin assembly for weak barbed-end capping are partly due to a depletion of the monomeric actin pool in the biomimetic assay [31], we also investigated a situation in which actin networks were initiated from a fixed concentration of G-actin. However, low concentrations of capping protein in the presence of high amounts of G-actin leads to an uncontrolled barbed-end assembly from WASp beads and the formation of nonpolarized actin networks whose fluorescence signal was difficult to quantify (S4C Fig [25,37]). The geometry of such actin networks also does not reflect the well-defined structure of the actin patches observed in the *cap1*Δ or *cap2*Δ cells (Fig 4F). Overall, the different conditions tested for these experiments (Fig 4C, 4D and 4E, S4A, S4B and S4C Fig) suggest that the pool of actin monomers reaches a deterministic steady state rapidly when experiments are initiated from F-actin and mimics best what is observed in cells.

### Effect of ADF/cofilin on Arp2/3-based and formin-based actin assembly pathways

Another protein that is expected to impact actin assembly is ADF/cofilin. While ADF/cofilin is mostly known for its importance on actin disassembly [2,13,14,31], it is also identified as a strong competitor of the Arp2/3 complex [38]. ADF/cofilin inhibits actin nucleation and branch formation by the Arp2/3 complex, but ADF/cofilin does not have, to our knowledge, any reported direct effect on actin nucleation or elongation by formins.

**Fig 5:**
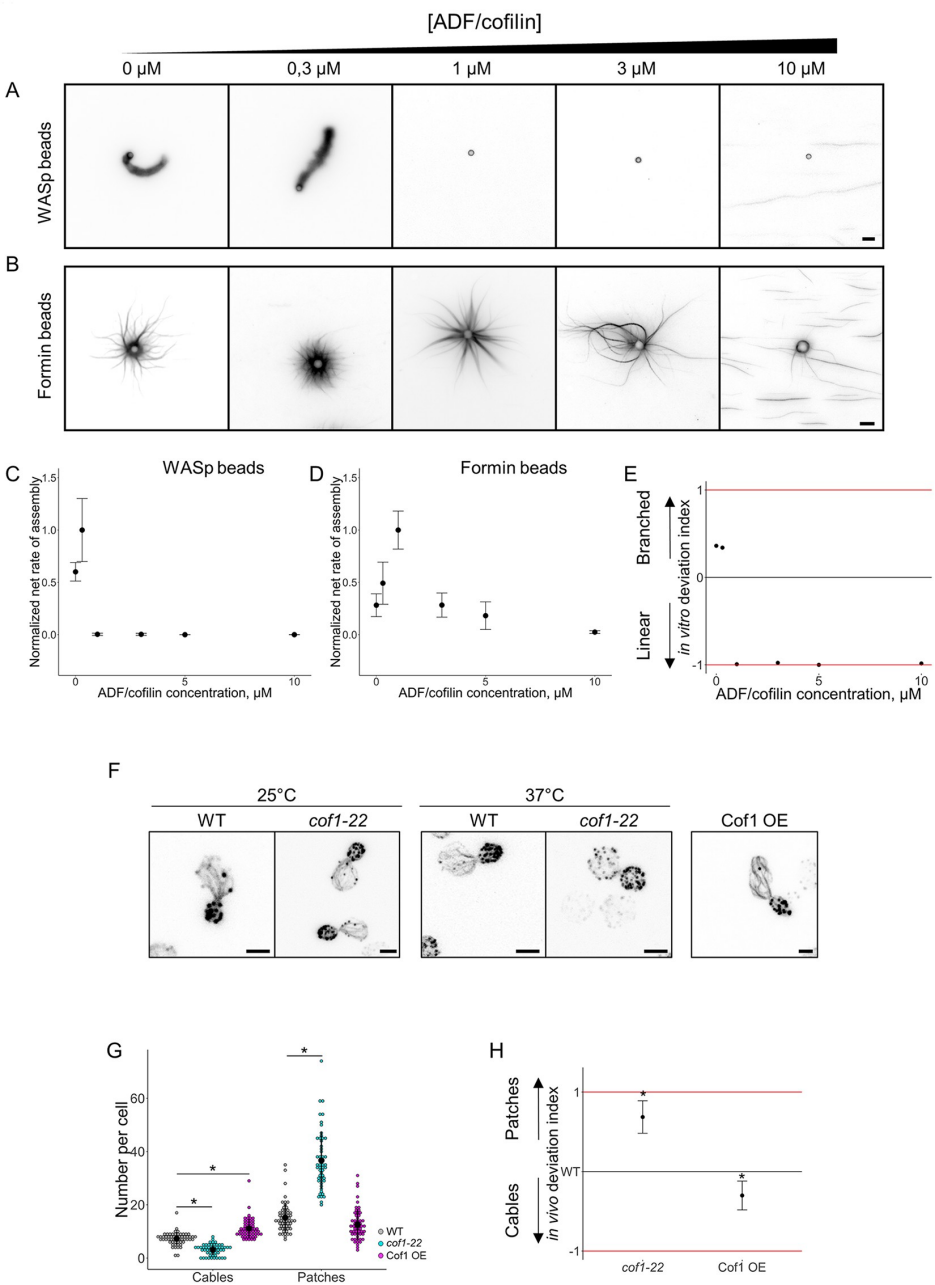
Modulation of the branched-to-linear actin network balance by ADF/cofilin. The underlying data can be found within S1 Data. A. Fluorescence snapshots of actin networks assembled around WASp-coated microbeads in the presence of fluorescent actin, Arp2/3 complex, profilin, capping protein, and variable concentrations of ADF/cofilin. Images were taken 30 min after the initiation of the experiment. Scale bar: 5 μm. B. Fluorescence snapshots of actin networks assembled around formin-coated microbeads in the presence of fluorescent actin, Arp2/3 complex, profilin, capping protein, and variable concentrations of ADF/cofilin. Images were taken 30 min after the initiation of the experiment. Scale bar: 5 μm. C. Quantification of (A). Net rate of actin assembly around WASp-coated microbeads as a function of the ADF/cofilin concentration, normalized to the maximum value. D. Quantification of (B). Net rate of actin assembly around formin-coated microbeads as a function of the ADF/cofilin concentration, normalized to the maximum value. E. In vitro deviation index, calculated as a function of the ADF/cofilin concentration. F. Snapshots of the actin cytoskeleton organization of budding yeast cells fixed and labeled with fluorescent phalloidin for ADF/cofilin overexpressing cells (right image) and for *cof1-22* cells at nonrestrictive (25 °C; left images) and restrictive (37 °C; center images) temperatures. Scale bars: 2 μm. G. Quantification of (F). Average number of actin patches and cables per cell in the wild-type, *cof1-22* mutant, and ADF/cofilin overexpressing conditions. H. In vivo deviation index of ADF/cofilin overexpressing cells and *cof1-22* cells at 37 °C. ADF, actin-depolymerizing factor; Arp2/3, actin-related protein 2/3; WASp, Wiskott–Aldrich syndrome protein.

ADF/cofilin at various concentrations ranging from 0 to 10 μM was added in the biomimetic assay, and the net rates of actin assembly were measured (Fig 5A and 5B). Similar to profilin, optimal concentrations of ADF/cofilin are different for the assembly of actin on WASp-coated beads (around 0.3 μM of ADF/cofilin) and for the assembly of actin on formin-coated breads (around 1 μM of ADF/cofilin) (Fig 5C and 5D). Low concentrations of ADF/cofilin favored the assembly of actin on WASp-coated beads. On the contrary, concentrations of ADF/cofilin above 1 μM reduced sharply actin assembly on WASp-coated beads, while actin assembly remained possible on formin-coated beads. Overall, the in vitro deviation index for ADF/cofilin indicates that while low concentrations of ADF/cofilin (<1 μM) favor branched network assembly over linear network assembly, higher concentrations of ADF/cofilin (>1 μM) progressively favor linear network assembly over branched network assembly (Fig 5E). We compared these results with the effect of an inhibition of a temperature-sensitive mutant of ADF/cofilin (*cof1-22*) [39] and of an overexpression of ADF/cofilin in yeast. As expected, while *cof1-22* cells did not show any visible defect in actin patch or cable assembly at nonrestrictive temperature (25 °C), *cof1-22* cells had an abnormally high number of patches and less cables at 37 °C. On the contrary, overexpressing cells had a small decrease of patch numbers and significantly more actin cables (Fig 5F and 5G and S2A Fig). Comparison of the in vivo and in vitro deviation indexes show a similar trend for ADF/cofilin and predicts that a higher concentration of ADF/cofilin in cells would progressively imbalance cells toward even more cable assembly (Fig 5E and 5H).

### Effect of tropomyosin on Arp2/3-based and formin-based actin assembly pathways

Finally, we tested the impact of tropomyosin in the biomimetic assay. Tropomyosin also strongly modulates actin assembly. First, as a competitor of the Arp2/3 complex, it inhibits actin nucleation and branch formation by the Arp2/3 complex [40–42]. Second, tropomyosin cooperates with profilin to enhance formin-dependent nucleation of actin cables [43].

**Fig 6:**
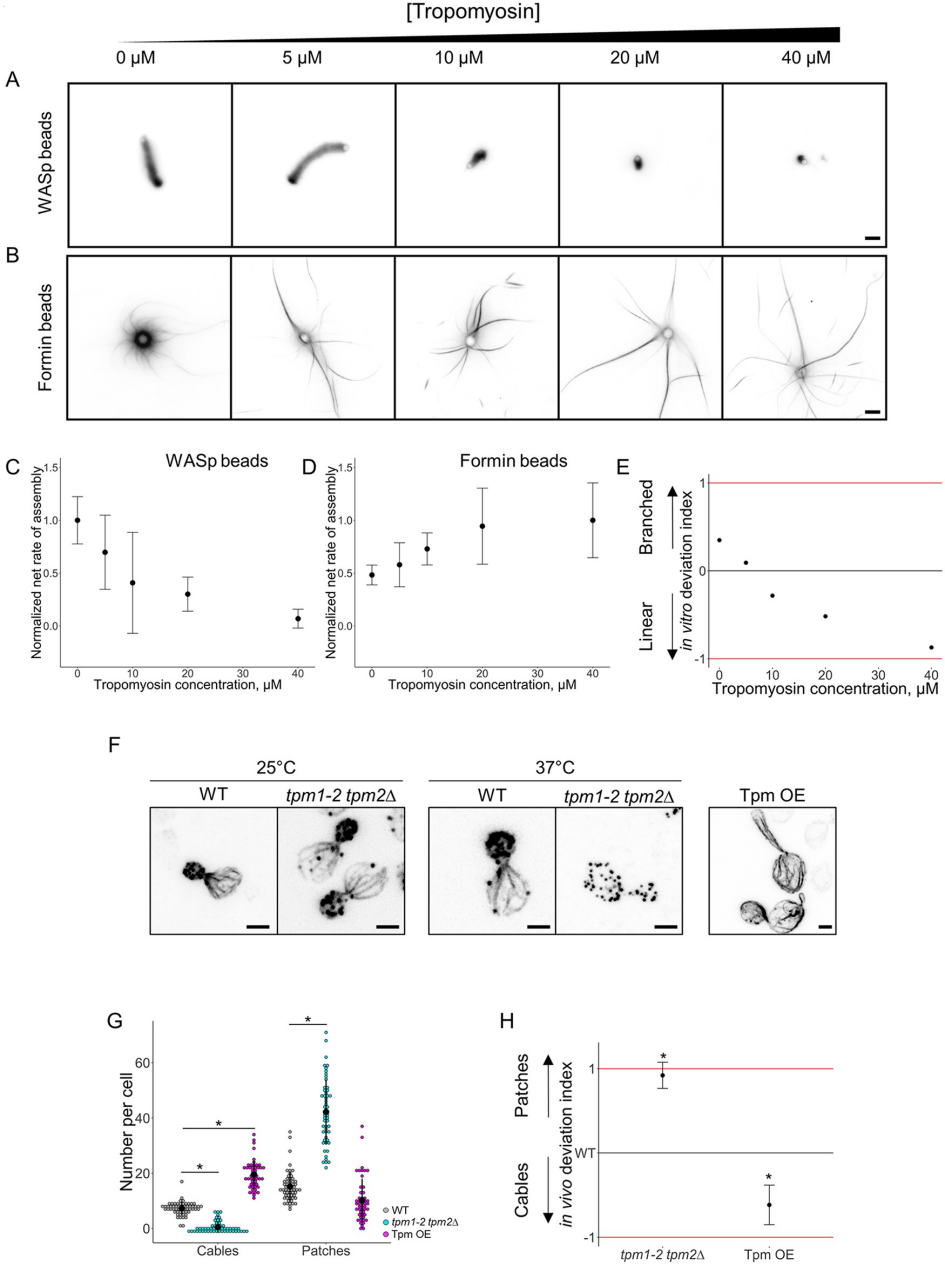
Modulation of the branched-to-linear actin network balance by tropomyosin. The underlying data can be found within S1 Data. A. Fluorescence snapshots of actin networks assembled around WASp-coated microbeads in the presence of fluorescent actin, Arp2/3 complex, profilin, capping protein, and variable concentrations of tropomyosin. Images were taken 30 min after the initiation of the experiment. Scale bar: 5 μm. B. Fluorescence snapshots of actin networks assembled around formin-coated microbeads in the presence of fluorescent actin, Arp2/3 complex, profilin, capping protein, and variable concentrations of tropomyosin. Images were taken 30 min after the initiation of the experiment. Scale bar: 5 μm. C. Quantification of (A). Net rate of actin assembly around WASp-coated microbeads as a function of the tropomyosin concentration, normalized to the maximum value. D. Quantification of (B). Net rate of actin assembly around formin-coated microbeads as a function of the tropomyosin concentration, normalized to the maximum value. E. In vitro deviation index, calculated as a function of the tropomyosin concentration. F. Snapshots of the actin cytoskeleton organization of budding yeast cells fixed and labeled with fluorescent phalloidin for tropomyosin overexpressing cells (right image) and for *tpm1-2 tpm2*Δ at nonrestrictive (25 °C; left images) and restrictive (37 °C; center images) temperatures. Scale bars: 2 μm. G. Quantification of (F). Average number of actin patches and cables per cell. H. In vivo deviation index of tropomyosin overexpressing cells and *tpm1-2 tpm2*Δ cells at 37°C. Arp2/3, actin-related protein 2/3; WASp, Wiskott–Aldrich syndrome protein.

Various concentrations of tropomyosin ranging from 0 to 40 μM were added in the biomimetic assay and we measured the net rates of actin assembly (Fig 6A and 6B). Tropomyosin progressively reduced the rate of actin assembly on WASp-coated beads, while it progressively increased actin assembly on formin-coated beads (Fig 6C and 6D). As a consequence, the in vitro deviation index predicts a shift from a favorable branched actin assembly in the absence of tropomyosin to a favorable actin cable assembly in the presence of increasing concentrations of tropomyosin (Fig 6E). We compared these results with the effect of an inhibition of a temperature-sensitive mutant of tropomyosin (*tpm1-2 tpm2*Δ) [44] and with an overexpression of tropomyosin in yeast. As previously reported, while *tpm1-2 tpm2*Δ cells did not show any visible defect in actin patch or cable assembly at nonrestrictive temperature (25 °C), *tpm1-2 tpm2*Δ cells presented a higher number of patches and a severe decrease of cables number at 37 °C. On the contrary, overexpression of tropomyosin induced a dramatic phenotype where cells are often misshaped, with a much-reduced number of actin patches and the formation of numerous actin cables (Fig 6F and 6G and S2A Fig). Side-by-side analysis of the in vitro and in vivo deviation indexes reveal a similar trend for both indexes (Fig 6E and 6H). A small shift between these indexes in the absence of tropomyosin also suggests a possible slight overestimation of cable stability in the bead assay at low concentration of tropomyosin (see Discussion).

## Discussion

**Fig 7:**
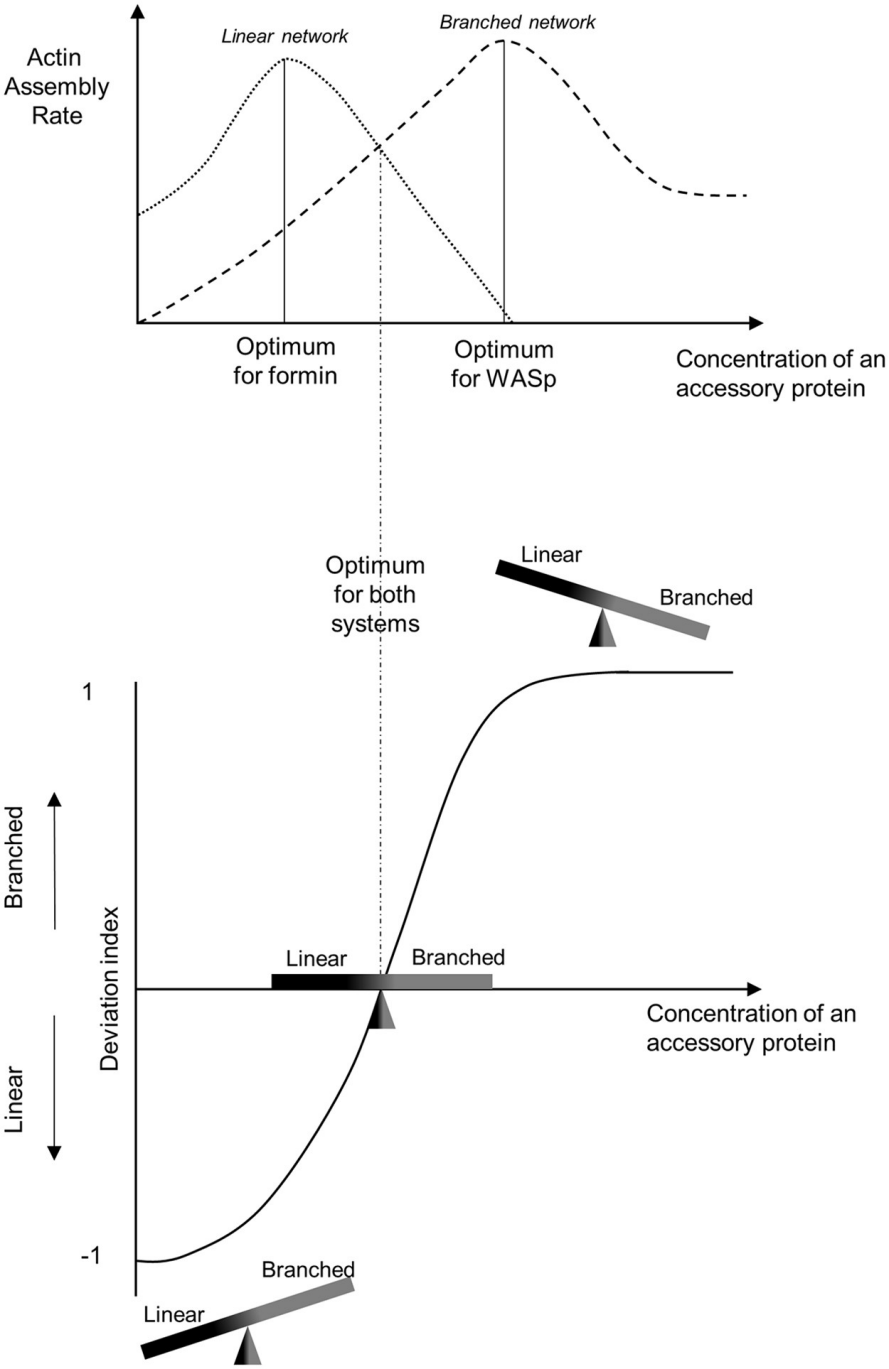
Cartoon representing how the size of actin networks is controlled in a common and competing environment. Results from this study indicate that actin assembly pathways (e.g., branched Arp2/3 and linear formin) do not have the same sensitivities to variable concentrations accessory proteins (upper panel). Principally, actin assembly rates vary differently and can span from cases in which only one type of network is able to assemble to cases in which they both assemble with similar efficiencies. Optimum concentrations of accessory proteins are also generally different for both pathways. Arp2/3, actin-related protein 2/3.

In this work, we measured quantitatively for a range of communal biochemical conditions the efficiencies of actin assembly on dually present branched (WASp/Arp2/3) networks and on linear (formin) networks. For most actin accessory proteins tested in this work, actin network assembly rates were strongly dependent on the concentration of the actin accessory proteins present in solution. Assembly rates displayed bell-shaped curves almost systematically, indicating the existence of optimal concentrations of these accessory proteins for the assembly of actin networks. The effect of these accessory proteins is different for the assembly properties of each actin networks (Fig 7, upper panel), which suggests that all accessory proteins control the amount of actin polymer assembled on each pathway. Actin regulators generally exhibited their characteristic differential effects on branched and linear networks even in the absence of competition for soluble factors. The main differences observed were that (1) an accessory protein may be essential for the assembly of an actin network but not another network and (2) that the range of optimal concentrations for actin assembly is not necessarily the same for both networks (Fig 7, upper panel).

Our results indicate that rates of actin assembly for each individual actin assembly pathway should not be interpreted separately. The existence of a global competition between actin networks requires an interpretation of these results in the light of how a common pool of components is shared in a given biochemical environment (Fig 7, lower panel).

Predicting the shape of these curves is difficult because accessory proteins often have multiple effects on actin assembly. However, our results indicate that in most cases, a side-by-side comparison of actin assembly rates between these structures is sufficient to predict which actin network will be favored in cells, i.e., in an environment in which the competition for a limiting pool of actin monomers exists (Fig 7, lower panel). If we compare now the effect of all the actin accessory proteins tested in this study, we notice that profilin is the most efficient regulator to switch actin assembly from branched to linear networks, i.e., in which the in vitro deviation index varies to its extreme values. Other accessory proteins also regulate actin network homeostasis but only partially.

We also verified the validity of our predictions by evaluating the numbers and intensities of actin patches and actin cables in yeast for a variety of mutant conditions. We generally found an exact correlation between the predictions made with the in vitro reconstitution assay and the observations in cells, indicating that actin assembly rules the balance between branched and linear networks for most accessory proteins. Such biomimetic system can also help us to make predictions that would be difficult to test experimentally. For example, our model predicts that an overexpression of Arp2/3 in cells would not prevent a remaining population of cables to assemble. In the absence of tropomyosin, we found that observations in the biomimetic overestimated slightly the number of actin cables. A possible explanation is that in the case of tropomyosin specifically, the size of actin structures may not be only determined by the effect of tropomyosin on actin assembly but may also be partially controlled by its effect on actin disassembly. This hypothesis is strongly supported by previous models, suggesting that tropomyosin protects actin filaments from the cellular actin disassembling machinery [45,46]. It would be interesting in the future to integrate principles of actin disassembly in the biomimetic assay developed in this study. For instance, the addition of factors such as Aip1 and coronin would determine to which extent actin disassembly may also regulate the size of actin networks in cells [47–49].

General principles highlighted in this work are likely to apply to other cellular models, although the existence of multiple overlapping actin structures in higher eukaryotes makes these principles harder to decipher. Notably, the inhibition or the depletion of the Arp2/3 complex leads to an excess of F-actin linear networks such as bundles and transverse arcs at the expense of lamellipodial networks and ruffle formation in insect and animal cells [50–53]. Conversely, profilin depletion increases the branching density and enhances Arp2/3 complex localization to the lamellipodium, whereas F-actin linear structures are strongly impaired [6]. Strikingly, microinjection of profilin in cells rescues the phenotype and induces the formation of F-actin bundles to the expense of the peripheral lamellipodia length [6]. Generally, ADF/cofilin and tropomyosin depletions cause the expansion of branched networks [11,54]. On the contrary, elimination of any of the tropomyosin isoforms fatally compromises stress fiber formation [45]. Unexpectedly, our results indicate that a low barbed-end capping activity favors actin assembly on branched networks in conditions in which the pool of polymerizable actin drops. This is likely due to the fact that nucleation by the Arp2/3 complex generates a large number of barbed ends, which remain free to elongate in the absence of capping. In an apparent contradiction, depletion of capping protein in many cell types abolishes the lamellipodium and promotes the formation of filopodial structures [28,55,56]. However, these data do not indicate to our knowledge whether actin filaments elongating in these structures are nucleated and elongated by formins or if they simply elongate due to the absence of sufficient capping in the lamellipodium.

As the size, shape, and activity of many other cellular structures are tightly regulated by a large number of factors in a competitive environment, we expect that a better understanding of their regulation will emerge from the principles presented in this work.

## Material and methods

### Plasmid constructions

#### Plasmids for protein expression and purification

Standard methods were employed for DNA manipulations. DNA fragments corresponding to gene coding sequences were obtained by PCR amplification (Phusion High-Fidelity DNA Polymerase, Finnzymes) of *Saccharomyces cerevisiae* genomic DNA. For WASp overexpression (Gst-Las17(375-Cter)-6xHis), the coding sequence was cloned in pGEX-4T-1 plasmid between BamHI and NotI. This construction of Las17 keeps the poly-L-proline domains necessary for the interaction with profilin. For capping protein overexpression (6xHis-Cap1/Cap2), the coding sequence of Cap1 was cloned in pRSFDuet-1 plasmid between BamHI and NotI and the coding sequence of Cap2 was cloned between BglII and XhoI. For tropomyosin overexpression, the coding sequence of Tpm1 was cloned in pRSFDuet-1 plasmid between NdeI and PacI with an Ala-Ser extension at the N-terminal end in order to increase actin affinity [57]. For formin overexpression (Gst-Bni1(1215-Cter)-TEV-9xHis, corresponding to the proline rich FH1 and the FH2 domains) the coding sequence of Gst and Bni1 were cloned simultaneously in a yeast multicopy plasmid (2 μ URA3) under the control of a GAL1 promoter between BamHI and PacI [48].

#### Plasmids for overexpression and phalloidin labeling

The coding sequences of profilin, ADF/cofilin, tropomyosin (Tpm1 and Tpm2 with an Ala-Ser extension), and capping protein (Cap1 and Cap2) were cloned in yeast 2 μ multicopy plasmids under the control of a GAL1 promoter. The plasmids are derived from the pRS425 and pRS426-based constructs that we use for protein purification in yeast, but a stop codon was kept at the end of the coding sequences. Control of capping protein overexpression was also verified with a 9 myc-tagged Cap2.

### Protein expression, purification and labeling

#### Actin

*S*. *cerevisiae* actin was purified from commercially purchased baker’s yeast (Kastalia, Lesaffre, Marcq-en-Baroeul, France), as described in [48,58]. As labeling on cysteines impacts profilin binding, rabbit muscle actin was purified and labeled on lysines with Alexa Succinimidyl Ester dyes, as described in [59,60].

#### Arp2/3 complex

*S*. *cerevisiae* Arp2/3 complex was purified from commercially purchased baker’s yeast (Kastalia, Lesaffre, Marcq-en-Baroeul, France) based on a protocol modified from [61,62]. Droplets of liquid yeast culture were frozen in liquid nitrogen and ground in a steel blender (Waring, Winsted, CT, USA). 50 g of ground yeast powder was mixed with a lysis buffer (20 mM Tris-HCl pH 7.5, 150 mM NaCl, 2 mM EDTA, 1 mM DTT supplemented with protease inhibitors (Set IV, Calbiochem, Merck4Biosciences, Darmstadt, Germany). The yeast extract was cleared by centrifugation at 160,000 g for 30 min and fractioned by a 50% ammonium sulfate cut. The insoluble fraction was dissolved and dialyzed in HKME buffer (25 mM Hepes pH 7.5, 50 mM KCl, 1 mM EGTA, 3 mM MgCl_2_, 1 mM DTT, 0,1 mM ATP) overnight at 4 °C. This fraction was then loaded onto a 2-ml Glutathione-Sepharose 4B (GE Healthcare Life Sciences, Piscataway, NJ, USA) column pre-charged with GST-N-WASp-VCA [61,62]. Bound Arp2/3 complex was purified with HKME buffer and eluted with 20 mM Tris-HCl pH 7.5, 25 mM KCl, 200 mM MgCl_2_, 1 mM EGTA and 1 mM DTT. Fractions of interest were detected by Bradford assay, pooled, concentrated with an Amicon Ultra 4-ml device (Merck4Biosciences, Darmstadt, Germany), and dialyzed against HKG buffer (20 mM Hepes, pH 7.5; 200 mM KCl; 6% glycerol).

#### Formin

*S*. *cerevisiae* formin was overexpressed in yeast (MATa, leu2, ura3-52, trp1, prb1-1122, pep4-3, pre1-451) under the control of a GAL1 promoter for 12 h at 30 °C with 2% galactose. Cells were harvested by centrifugation, frozen in liquid nitrogen, and ground in a steel blender (Waring, Winsted, CT, USA). For protein purification, 5 g of ground yeast powder was mixed with 45 ml of HKI10 buffer (20 mM Hepes, pH 7.5; 200 mM KCl; 10 mM Imidazole, pH 7.5), supplemented with 50 μl of protease inhibitors (Set IV, Calbiochem, Merck4Biosciences, Darmstadt, Germany), and thawed on ice. The mixture was centrifugated at 160,000 g for 30 min, and the supernatant was incubated with 500-μl bed volume of Nickel-Sepharose 6 Fast Flow (GE Healthcare Life Sciences, Piscataway, NJ, USA) for 2 h at 4 °C. Bound protein was batch purified with HKI20 buffer (20 mM Hepes, pH 7.5; 200 mM KCl; 20 mM Imidazole, pH 7.5) and was TEV-cleaved from Nickel-Sepharose for 1 h at room temperature. The protein was concentrated with an Amicon Ultra 4 ml device (Merck4Biosciences) and dialyzed against HKG buffer.

#### WASp and capping protein

*S*. *cerevisiae* WASp were overexpressed in Rosetta 2(DE3)pLysS cells. Bacteria were lysed in 20 mM Tris-HCl pH 7.5, 1 mM DTT, 1 mM EDTA, 200 mM NaCl, 0,1% Triton X-100, 5% glycerol and protease inhibitors (Complete Protease Inhibitor Cocktail, Roche). After clearing the bacterial lysate by centrifugation at 160,000 g for 20 min, the protein was subjected to a first-step purification on Glutathione-Sepharose beads following the manufacturer’s recommendations. It was then eluted with 100 mM L-glutathione reduced and further purified by the addition of Nickel-Sepharose beads 6 Fast Flow (GE Healthcare Life Sciences, Piscataway, NJ, USA). An elution was performed using HKI500 buffer (20 mM Hepes, pH 7.5; 200 mM KCl; 500 mM Imidazole, pH 7.5). The protein was concentrated with an Amicon Ultra 4-ml device (Merck4Biosciences) and dialyzed against HKG buffer.

*S*. *cerevisiae* capping protein was purified with a similar protocol including only a Nickel-Sepharose purification step in HKI20 buffer (20mM Hepes pH 7.5, 200 mM KCl, 20 mM imidazole pH 7.5, 0,1% Triton X-100, 10% glycerol).

#### Tropomyosin

*S*. *cerevisiae* tropomyosin was overexpressed in Rosetta 2(DE3)pLysS cells and purified based on a protocol modified from [63]. Briefly, cells were lysed in extraction buffer (50 mM imidazole-HCl, pH 6.9, 300 mM KCl, 5 mM MgCl_2_, 0.3 mM phenylmethylsulfonyl fluoride and protease inhibitors (Complete Protease Inhibitor Cocktail, Roche)) by sonication and boiled for 10 min. Cell debris and insoluble proteins were pelleted at 300,000 g for 20 min. The clear supernatant containing pure tropomyosin was finally dialyzed into 50 mM KCl, 10 mM Tris-HCl pH 7.5 and 0.5 mM DTT overnight at 4 °C.

#### Profilin and ADF/cofilin

*S*. *cerevisiae* profilin and cofilin were overexpressed in Rosetta 2(DE3)pLysS cells and purified as described in [48,64,65].

### Actin assembly assays

#### Functionalization of beads

Polystyrene microspheres (2 μm diameter, 2.5% solids [w/v] aqueous suspension, Polysciences, Inc) were diluted 10 times in HK buffer (20 mM Hepes pH 7.5, 150 mM KCl) and incubated with 100 nM Las17 for 30 min on ice. Beads were saturated with 1% bovine serum albumin (BSA) for 15 min, washed and eventually stored on ice in HK buffer supplemented with 0,1% BSA. Bni1 (1 μM) was coated on glutathione-coated particles (4.37 μm diameter, 0.5% solids [w/v] aqueous suspension, Spherotech, Inc) with a similar protocol.

#### Bead motility assays

Unlabeled and labeled actins were mixed to reach a final labeling percentage of 2%. Actin was prepolymerized in KMEI buffer (50 mM KCl, 1 mM MgCl_2_, 1 mM EGTA, 10 mM Imidazole pH 7.8) for 1 h at room temperature. To initiate actin network assembly, Las17 and Bni1-coated beads (10^4^ beads of each type per μl) were incubated with F-actin and other proteins in a motility buffer (20 mM Hepes pH 7.5; 100 mM KCl; 2 mM EGTA; 2 mM MgCl_2_; 50 mM DTT; 5 mM ATP; 0.3 mg/ml glucose; 0.03 mg/ml catalase; 0.15 mg/ml glucose oxidase; 0.8% methylcellulose 1,500 cP and 0.5% BSA). Standard optimal protein concentrations are 8 μM F-actin, 15 μM profilin, 1 μM capping protein, and 250 nM Arp2/3 complex unless otherwise stated. These values were kept as reference values for all experiments related to the titration of accessory proteins. For every titration experiment, the range of concentration of the tested protein was chosen so that its effect could be analyzed from its absence up to saturating amounts for both actin networks. Highest concentrations therefore represent situations that are out of the physiological regime.

#### Image acquisition, processing and analysis

For in vitro actin assembly assays, images were acquired on a Zeiss Axio Observer Z1 microscope equipped with a 100x/1.4NA Oil Ph3 Plan-Apochromat objective and a Hamamatsu ORCA-Flash 4.0LT camera. Images were acquired with Zen 2.3 blue edition.

#### Data quantification

All set of images were taken using the same light intensity and exposure time. Intensity values were measured over time before reaching the steady state using Fiji (Version 1.52e). Fluorescence of the background was subtracted for each value. Actin assembly rates r were calculated as follows:

$$r=\frac{I_{t_{2}}-I_{t_{1}}}{t_{2}-t_{1}},$$

in which I is the intensity value at a given time point t. Actin assembly rates were normalized to their maximal values. Data were quantified and statistically analyzed with GraphPad Prism 6.05, and plotted with R using the package ggplot2 (https://ggplot2.tidyverse.org). For all conditions, experiments were repeated independently at least 3 times. Data presented in the manuscript correspond to one set of experiments, which includes a minimum of *n* = 30 data points per condition from a minimum of 6 independent beads. In all plots of actin assembly rates, error bars indicate standard deviations.

### Actin organization in yeast

#### Yeast cell fixation and phalloidin staining

Yeast strains used in this study were obtained from previous published studies, including [21,36,39,44,66]. To assess actin organization, yeast cells were fixed and stained with fluorescently labeled phalloidin as described in [43,67]. Briefly, strains were grown in YPD medium at 25 °C to early/mid log phase and fixed at 25 °C with 4% formaldehyde for 1 h. Thermosensitive mutants were cultivated for 1 h at 37 °C before fixation. For protein overexpression, wild-type and cells carrying the plasmid of interest were grown in synthetic yeast medium supplemented with 2% galactose before fixation. Cells were then stained overnight at 25 °C with AlexaFluor-568-phalloidin and washed three times with PBS before imaging.

#### Cell imaging

Cells were imaged in PBS– 70% glycerol on a Leica TCS SP8 STED inverted confocal microscope using a 63x, 1.4 NA oil Plan Apochromatic objective lens in combination with a hybrid detector. Full z-stacks were acquired with LAS X software.

#### Data quantification

Number of actin cables and patches was scored from a maximal intensity projection done with Fiji. For all conditions, a minimum of 50 cells were imaged and analyzed. In all plots, error bars indicate standard deviations and symbols * indicate significant statistical differences compared to the wild-type conditions using a Student *t* test (*p* < 0.0001). All data used to draw conclusions are available in S1 Data.

## Supporting information

S1 Data Data for Figs 2, 3, 4, 5 and 6 and S1, S2, S3 and S4 Figs. (XLSX)

S1 Fig Fluorescence intensity analysis of branched and linear actin networks. The underlying data can be found within S1 Data. A. Fluorescence intensity of F-actin networks were quantified over time for standard conditions (in black) and for the most extreme perturbations performed (lowest protein concentration in green; highest protein concentration in red). Lines indicate linear regressions. B. Rate of actin assembly around 0.5-μm diameter WASp-coated microbeads in the presence of fluorescent actin, profilin, and capping protein as a function of the Arp2/3 complex concentration, normalized to the maximum value. Arp2/3, actin-related protein 2/3; F-actin, filamentous actin; WASp, Wiskott–Aldrich syndrome protein.
(TIF)

S2 Fig Wide field snapshots of the actin cytoskeleton organization in yeast strains presented in this study. Scale bars: 5 μm. The underlying data can be found within S1 Data. A. Budding yeast cells fixed and labeled with fluorescent phalloidin at the indicated temperatures. B. Quantification of Fig 2E based on total intensities and not numbers of actin structures. C. In vivo deviation index, based on structures intensities, calculated in the presence of DMSO and 200 μM CK-666. CK-666, Arp2/3 complex inhibitor I.
(TIF)

S3 Fig Branched and linear actin networks emerging from bead surfaces do not influence each other in these reconstituted assays. The underlying data can be found within S1 Data. A. Rate of actin assembly around WASp-coated microbeads as a function of the profilin concentration when formin-coated beads are not present. B. Rate of actin assembly around formin-coated microbeads as a function of the profilin concentration when WASp-coated beads are not present. C. In vitro deviation index, calculated as a function of the profilin concentration, measured from data obtained in (A) and (B). WASp, Wiskott–Aldrich syndrome protein.
(TIF)

S4 Fig Modulation of the branched-to-linear actin network balance by capping protein at steady state and in conditions in which actin assembly is initiated from G-actin. Quantification of actin networks assembly at steady-state around WASp-coated and formin-coated microbeads in the presence of fluorescent actin, Arp2/3 complex, profilin, and variable concentrations of capping protein. Left plots indicate rates of actin assembly around WASp-coated and formin-coated microbeads as a function of the capping protein concentration, normalized to the maximum value. Right plot indicates the in vitro deviation index calculated as a function of the capping protein concentration. The underlying data can be found within S1 Data. A. Condition in which the accessory proteins were incubated for 2 h at room temperature with prepolymerized actin (F-actin) before addition of the microbeads. B. Condition in which 8 μM G-actin, 15 μM profilin, and 250 nM Arp2/3 were incubated for 2 h at room temperature before addition of the microbeads. C. Condition in which 4 μM of G-actin, 12 μM of profilin, 250 nM Arp2/3, and the microbeads were incubated at room temperature simultaneously. Red dots indicate that the intensity of actin networks was difficult to quantify due to the uncontrolled barbed-end assembly around WASp-coated beads at low concentration of capping protein. The image is a fluorescence snapshot of an actin network assembled around WASp-coated microbeads in the presence of 4 μM fluorescent G-actin, 250 nM Arp2/3 complex, 12 μM profilin, and 100 nM capping protein, taken 30 min after the initiation of the experiment. Scale bar: 5 μm. Arp2/3, actin-related protein 2/3; F-actin, filamentous actin; G-actin, globular actin; WASp, Wiskott–Aldrich syndrome protein.
(TIF)

S5 Fig Overexpression of capping protein in yeast. Western blot control of capping protein overexpression with a 9 myc-tagged Cap2. Pgk1 is a loading control. Cap2, capping protein 2.
(TIF)

## Abbreviations

ABP: actin-binding protein
ADF: actin-depolymerizing factor
ADP: adenosine diphosphate
Arp2/3: actin-related protein 2/3
ATP: adenosine triphosphate
Bni1p: bud neck involved protein 1
Cap1/2: capping protein 1 and 2
CK-666: Arp2/3 complex inhibitor I
F-actin: filamentous actin
FH1-FH2: formin homology domain 1 and 2
G-actin: globular actin
Las17p: budding yeast WASp ortholog
Pfy1: profilin
WASp: Wiskott–Aldrich syndrome protein
