# Supplementary figures and images for: Sizes of actin networks sharing a common environment are determined by the relative rates of assembly
Source: PLoS Biol. 2019 Jun 10;17(6):e3000317. doi: 10.1371/journal.pbio.3000317 (PMC6586355; doi:10.1371/journal.pbio.3000317)

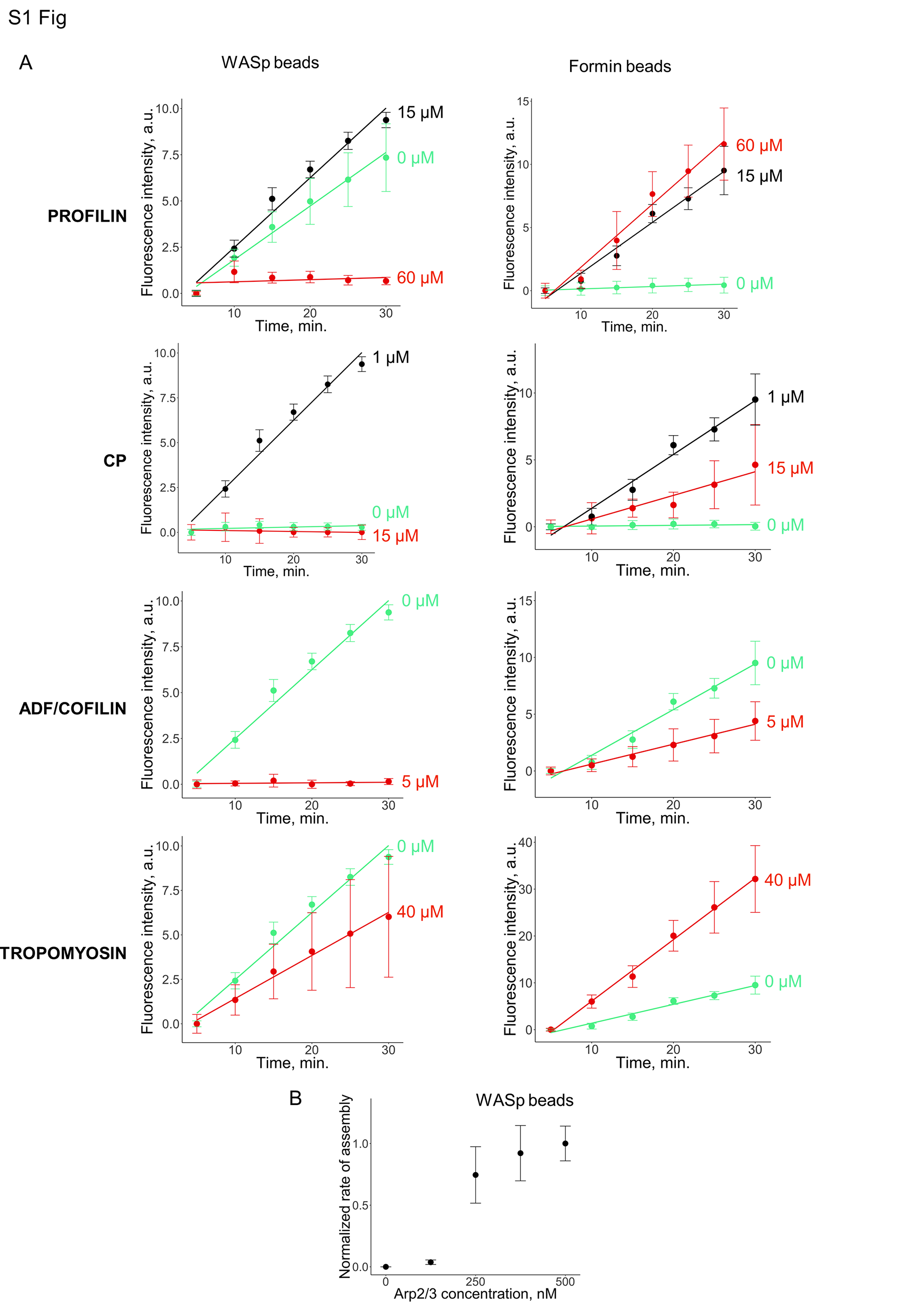

Supplement: S1 Fig — The underlying data can be found within S1 Data. A. Fluorescence intensity of F-actin networks were quantified over time for standard conditions (in black) and for the most extreme perturbations performed (lowest protein concentration in green; highest protein concentration in red). Lines indicate linear regressions. B. Rate of actin assembly around 0.5-μm diameter WASp-coated microbeads in the presence of fluorescent actin, profilin, and capping protein as a function of the Arp2/3 complex concentration, normalized to the maximum value. Arp2/3, actin-related protein 2/3; F-actin, filamentous actin; WASp, Wiskott–Aldrich syndrome protein. (TIF) [file pbio.3000317.s002.tif]

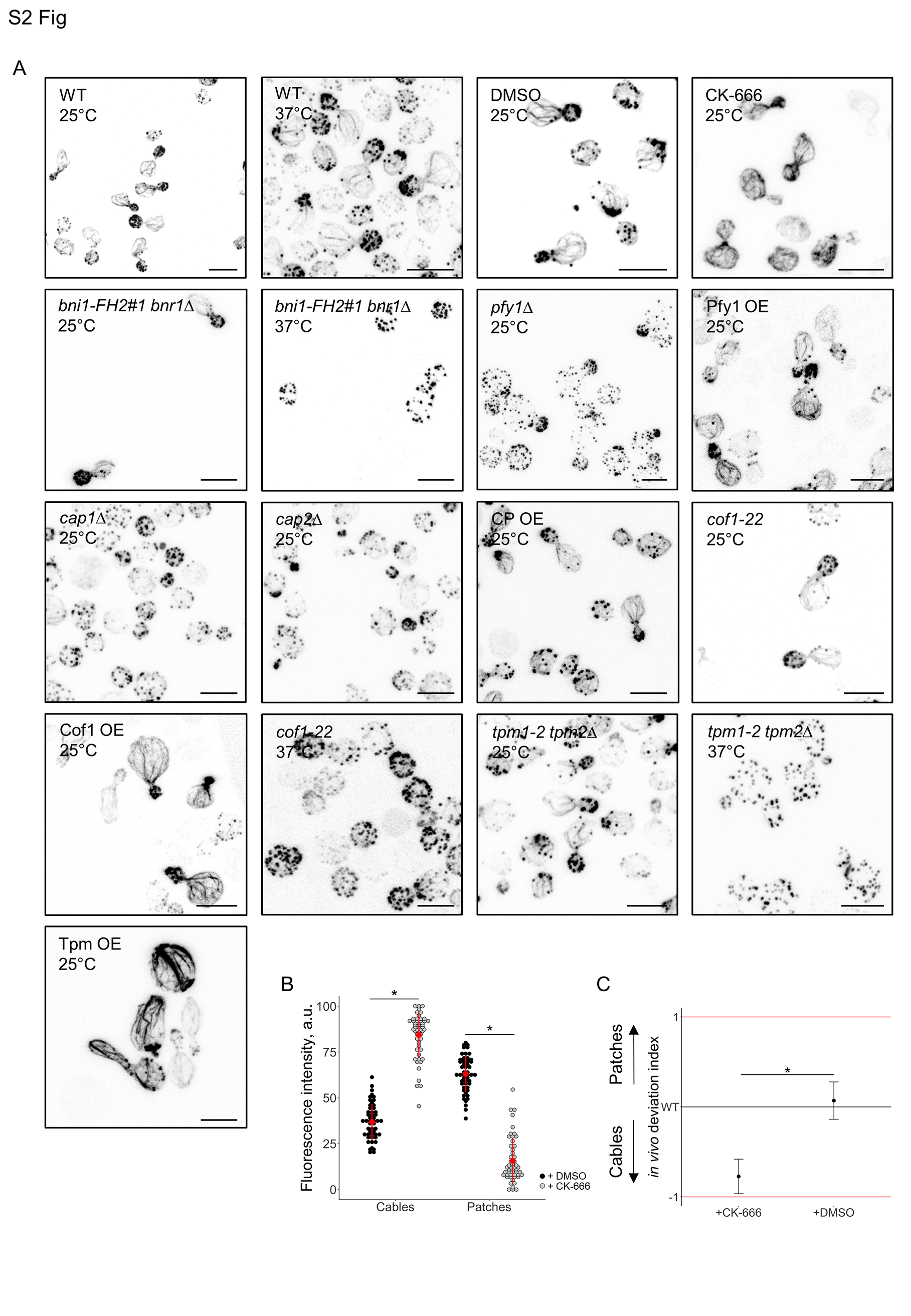

Supplement: S2 Fig — Scale bars: 5 μm. The underlying data can be found within S1 Data. A. Budding yeast cells fixed and labeled with fluorescent phalloidin at the indicated temperatures. B. Quantification of Fig 2E based on total intensities and not numbers of actin structures. C. In vivo deviation index, based on structures intensities, calculated in the presence of DMSO and 200 μM CK-666. CK-666, Arp2/3 complex inhibitor I. (TIF) [file pbio.3000317.s003.tif]

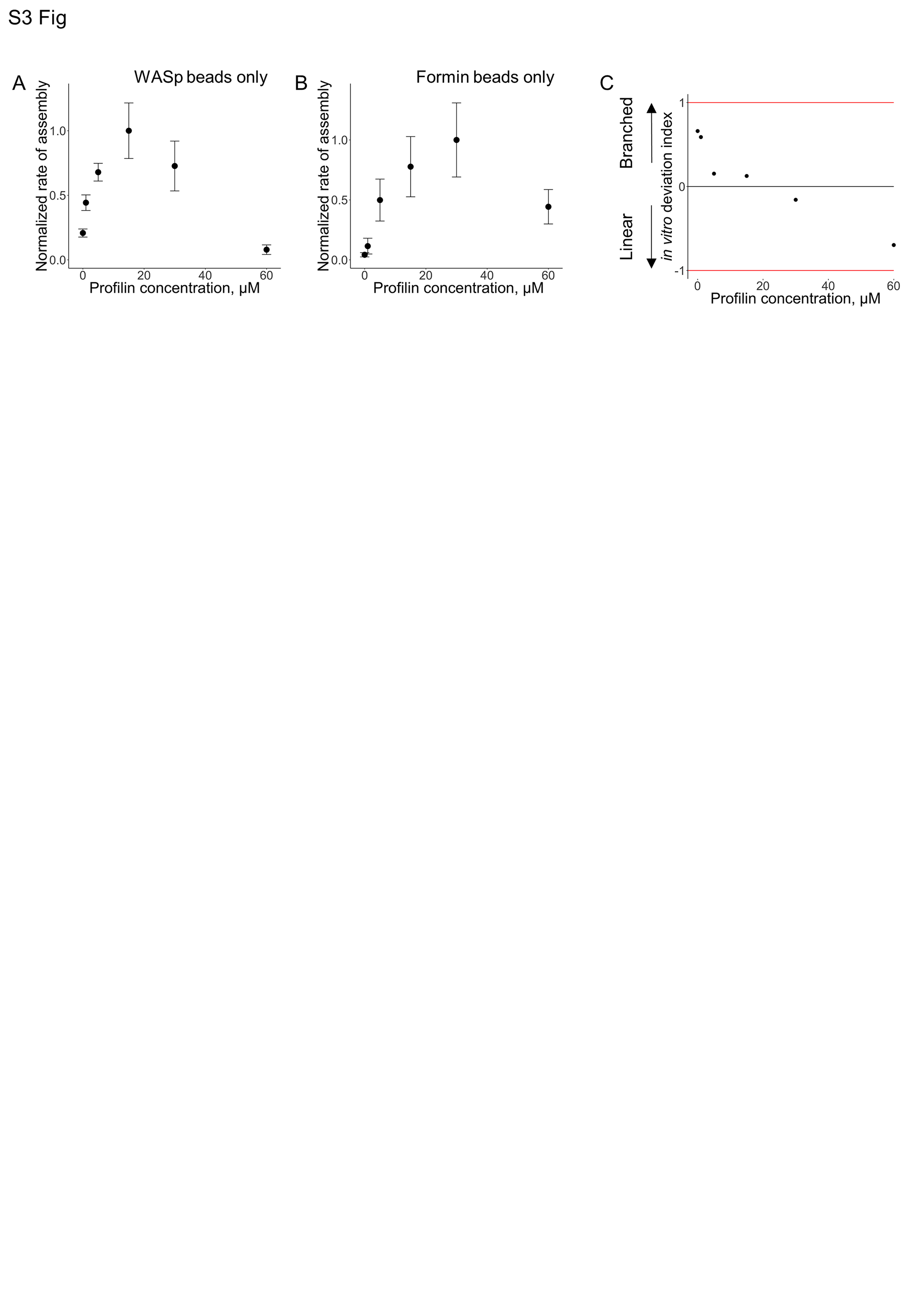

Supplement: S3 Fig — The underlying data can be found within S1 Data. A. Rate of actin assembly around WASp-coated microbeads as a function of the profilin concentration when formin-coated beads are not present. B. Rate of actin assembly around formin-coated microbeads as a function of the profilin concentration when WASp-coated beads are not present. C. In vitro deviation index, calculated as a function of the profilin concentration, measured from data obtained in (A) and (B). WASp, Wiskott–Aldrich syndrome protein. (TIF) [file pbio.3000317.s004.tif]

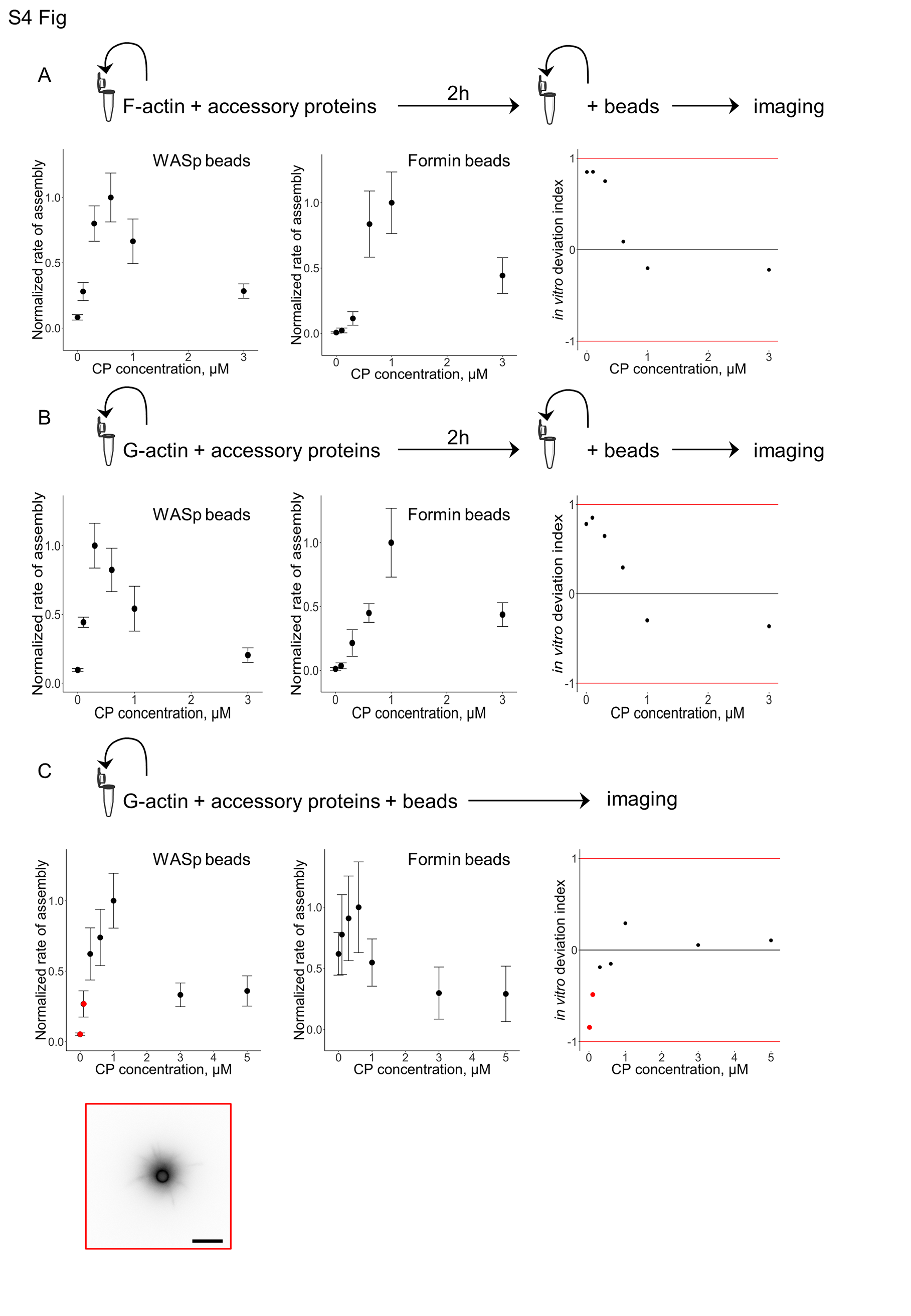

Supplement: S4 Fig — Quantification of actin networks assembly at steady-state around WASp-coated and formin-coated microbeads in the presence of fluorescent actin, Arp2/3 complex, profilin, and variable concentrations of capping protein. Left plots indicate rates of actin assembly around WASp-coated and formin-coated microbeads as a function of the capping protein concentration, normalized to the maximum value. Right plot indicates the in vitro deviation index calculated as a function of the capping protein concentration. The underlying data can be found within S1 Data. A. Condition in which the accessory proteins were incubated for 2 h at room temperature with prepolymerized actin (F-actin) before addition of the microbeads. B. Condition in which 8 μM G-actin, 15 μM profilin, and 250 nM Arp2/3 were incubated for 2 h at room temperature before addition of the microbeads. C. Condition in which 4 μM of G-actin, 12 μM of profilin, 250 nM Arp2/3, and the microbeads were incubated at room temperature simultaneously. Red dots indicate that the intensity of actin networks was difficult to quantify due to the uncontrolled barbed-end assembly around WASp-coated beads at low concentration of capping protein. The image is a fluorescence snapshot of an actin network assembled around WASp-coated microbeads in the presence of 4 μM fluorescent G-actin, 250 nM Arp2/3 complex, 12 μM profilin, and 100 nM capping protein, taken 30 min after the initiation of the experiment. Scale bar: 5 μm. Arp2/3, actin-related protein 2/3; F-actin, filamentous actin; G-actin, globular actin; WASp, Wiskott–Aldrich syndrome protein. (TIF) [file pbio.3000317.s005.tif]

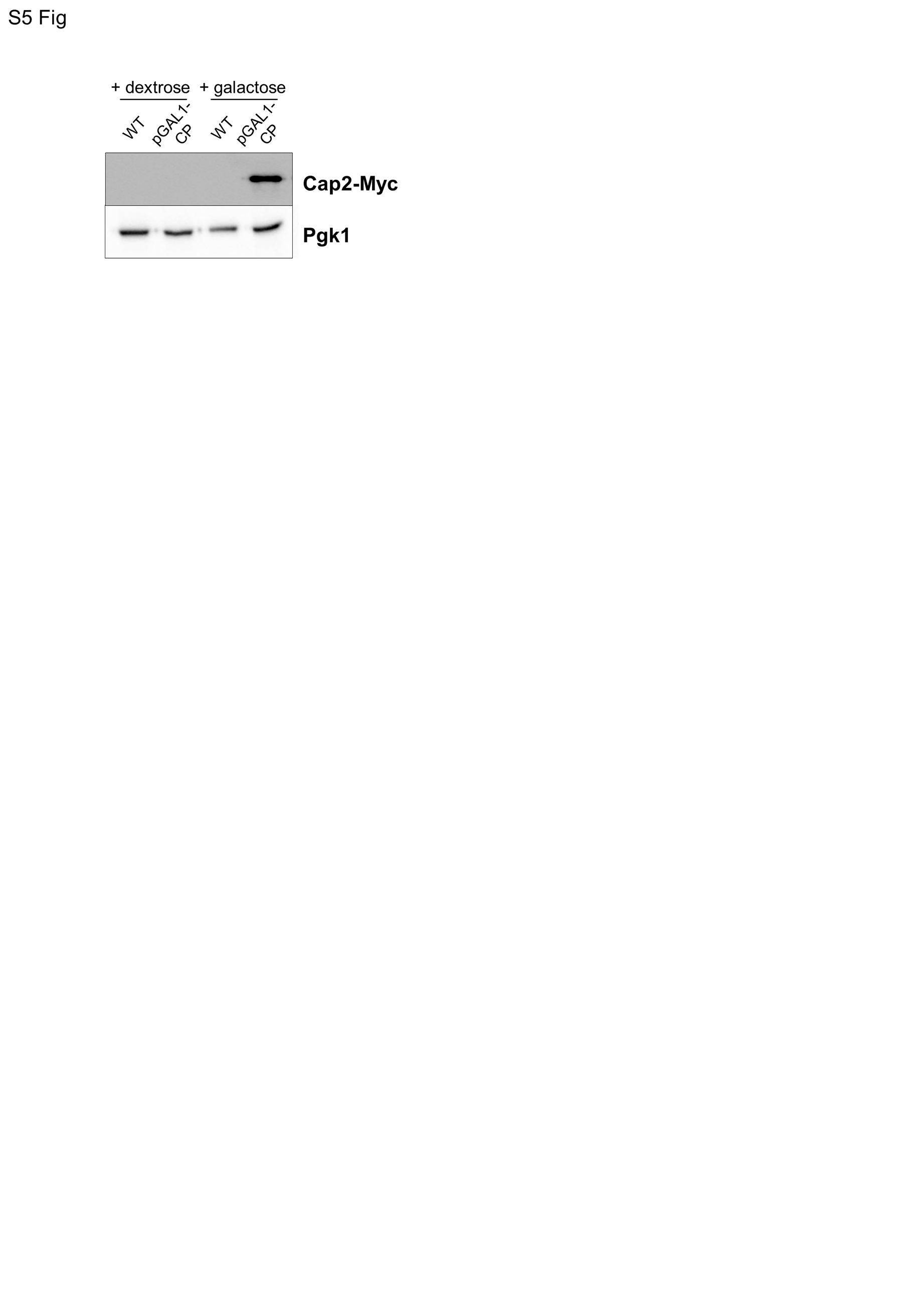

Supplement: S5 Fig — Western blot control of capping protein overexpression with a 9 myc-tagged Cap2. Pgk1 is a loading control. Cap2, capping protein 2. (TIF) [file pbio.3000317.s006.tif]
